# Isthmus Dependent Atrial Flutter Cycle Length Correlates with Right Atrial Cross-Sectional Area

**Published:** 2009-05-15

**Authors:** Kousik Krishnan, Akshay Gupta, Sean M Halleran, Dave Chawla, Elizabeth F Avery, Julia L Bienias, Richard G Trohman

**Affiliations:** 1 Department of Internal Medicine, Section of Cardiology (Cardiac Electrophysiology Service); 2 Department of Preventive Medicine, Section of Biostatistics and Epidemiology, Rush University Medical Center, Chicago, IL

**Keywords:** Atrial Flutter, Reentrant arrhythmias, Catheter Ablation, Electrophysiology Study, Echocardiography, Right Atrium

## Abstract

**Background:**

Right atrial flutter cycle length can prolong in the presence of antiarrhythmic drug therapy. We hypothesized that the cycle length  of right atrial isthmus dependent flutter would correlate with right atrial cross-sectional area measurements.

**Methods:**

60 patients who underwent ablation for electrophysiologically proven isthmus dependent right atrial flutter, who were not on Class I or Class III antiarrhythmic drugs and had recent 2-dimensional echocardiographic data comprised the study group. Right atrial length and width were measured in the apical four chamber view. Cross-sectional area was estimated by multiplying the length and width. 35 patients had an atrial flutter rate ≥ 250 bpm (Normal Flutter Group) and 25 patients had an atrial flutter rate < 250 bpm (Slow Flutter Group).

**Results:**

Mean atrial flutter rate was 283 bpm in the normal flutter group and 227 bpm in the slow flutter group. Mean atrial flutter cycle length was 213 ms  in the Normal Flutter Group and 265 ms in the Slow Flutter Group (p< 0.0001). Mean right atrial cross sectional area was 1845 mm^2^ in the Normal Flutter group and 2378 mm^2^ in the Slow Flutter Group, (p< 0.0001). Using linear regression, CSA was a significant predictor of cycle length (β  =0.014 p = 0.0045). For every 1 mm^2^ increase in cross-sectional area, cycle length is 0.014 ms longer.

**Conclusions:**

In the absence of antiarrhythmic medications, right atrial cross sectional area enlargement correlates with atrial flutter cycle length. These findings provide further evidence that historical rate-related definitions of typical isthmus dependent right atrial are not mechanistically valid.

## Introduction

The classic definition of atrial flutter includes an atrial rate of 250-350 beats per minute (bpm) without an isoelectric baseline between atrial deflections [1]. It is well known that right atrial flutter cycle length can prolong in the presence of antiarrhythmic medication. Strong sodium channel blockers (flecainide, propafenone) and potassium channel blockers (e.g. ibutilide) decrease trans-isthmus conduction velocity or significantly increase the atrial refractory period. Ibutilide has a strong effect on the atrial mean action potential duration. Amiodarone has both sodium and potassium channel inhibitory effects, however, its impact on both conduction velocity and action potential duration is relatively weaker [2-5]. Little data exists correlating right atrial size with atrial flutter cycle length. One small study correlated acute volume expansion of the right atrium (by negative tilt and inspiration) with atrial flutter cycle length prolongation [6]. We hypothesized that right atrial isthmus dependent flutter cycle length would correlate with right atrial cross-sectional area (CSA) measurements.

## Methods

114 consecutive patients who underwent ablation of a right atrial isthmus dependent tachycardia and were not on Class I or Class III antiarrhythmic drugs were screened. All 114 patients had an electrophysiologically proven reentrant arrhythmia with the cavotricuspid isthmus (CTI) as part of the circuit. Of these patients, 60 who had recent 2D echocardiograms were included. The echocardiograms were performed when the patients were in atrial flutter. Right atrial flutter cycle lengths were measured and atrial rates were dichotomized to greater than or equal to 250 bpm or less than 250 bpm. Right atrial length and width dimensions were measured in the apical four chamber view at end diastole and a CSA calculation was made by multiplying the length and width.

### Statistical analysis

All data are expressed as mean ± SD unless otherwise noted. The unpaired 2-tailed t test was used to compare continuous variables between the two flutter groups. Linear regression was used to investigate the association between cycle length and CSA. A p-value of < 0.05 was considered significant.

## Results

In the group as a whole, mean tachycardia cycle length was 234.9 ±  31.5 ms.  The overall mean atrial flutter rate was 260 ±  35.2 bpm. The overall mean cross sectional area was 2066.9 ±  815.4  mm^2^.The flutter cycle length was 213 ms  in the Normal Flutter Group and 265 ms in the Slow Flutter Group (p< 0.0001). As seen in Table 1, there was a significant difference between slow and normal atrial flutters in both cycle length and cross sectional area.

**Figure 1:**
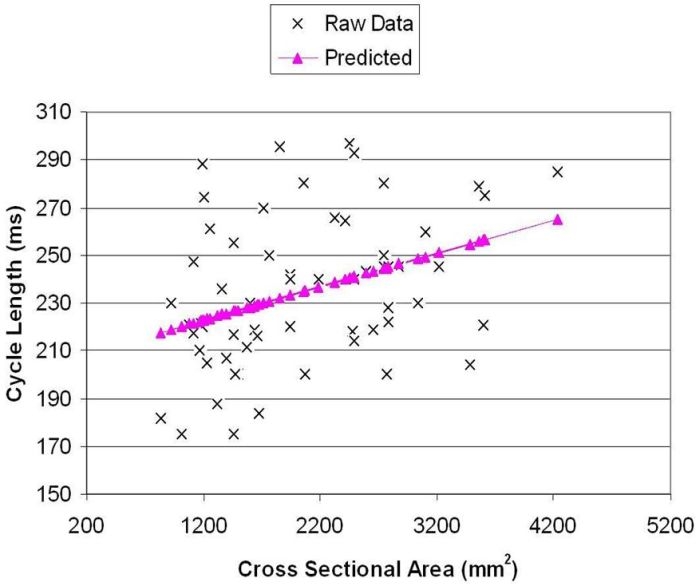
Cycle length vs cross sectional area. The scatter that occurs reflects the fact that CSA accounts for 13% of  atrial flutter cycle length and provides indirect proof that other influences such as scarring and anisotrpy are not linear correlates of CSA

We modeled cycle length as a function of CSA using linear regression.  The parameter estimate,  β for CSA was 0.014 (s.e. = 0.005, p  = 0.0045, R^2^ = .131),  such that one additional mm^2^ of cross sectional area was associated with a 0.014 ms longer cycle length ( a 500 mm^2^  increase in  right atrial area  would be associated with a 7  ms increase cycle length, see Figure 1). There was one patient with a very high CSA of 4234 mm^2^. As a sensitivity analysis, we re-fit the model without this person's data; the effect was still significant and β was slightly reduced, to 0.013 (R^2^ =.100), thus demonstrating that CSA had a 13% influence on the overall flutter cycle length.

## Discussion

For many years, definitions of atrial tachyarrhythmias were based solely on rate, with little mention of electrophysiological mechanisms. Atrial rates over 250 bpm were defined as flutter and rates less than 250 bpm were classified as atrial tachycardia, implying a link between rate and arrhythmia mechanism. This definition was revised in 2001, by the Joint Expert Group of the European Society of Cardiology and the Heart Rhythm Society, to eliminate the connection between the atrial rate and the arrhythmia mechanism. Their proposal was to term any regular atrial rhythm ≥ 100 bpm originating outside the sinus node region as an atrial tachycardia, whether the mechanism is focal or macroreentrant [7].

Isthmus-dependant atrial flutter is more easily cured with less recurrence than atrial tachycardias [8-13]. If decisions to offer catheter ablation are based strictly on rate criteria (rather than mechanism), our data suggests that a significant number of patients (25/60, 42%) might be excluded. These findings also emphasize the importance of careful diagnostic electrophysiological testing, mapping of activation sequences and pacing to identify concealed entrainment prior to delivery of ablative lesions. Our data corroborates recommendations of The Joint Expert Group of the European Society of Cardiology and the Heart Rhythm Society that atrial arrhythmia definitions should be mechanistic and demonstrates that an enlarged right atrial CSA correlates strongly with atrial flutter cycle length in the absence of antiarrhythmic drug therapy.

Although there is a correlation between atrial flutter cycle length and right atrial size, this relationship is not always linear and does not fully explain cycle length variation in typical atrial flutter. Cycle length is calculated by dividing the distance a wavefront of activation travels, D, by the wavefront's conduction velocity, V (Cycle length = D/V). It is well known that atrial tissue, via the distribution of connexins, prefers longitudinal anisotropic conduction compared to lateral conduction. This preference is even more pronounced than in ventricular tissue [14]. Conduction velocity is affected by cell size, excitability, as well as gap junction (connexin) location and density [15-17]. When the atria dilate due to volume overload, Connexin 43 redistributes within atrial cells to a lateral location, decreasing the normal longitudinal conduction [18]. In addition, fibrosis of the atrial myocardium plays an important role in gap junction disorganization Uncoupling of cellular gap junctions (by atrial fibrosis and dilatation) plays a much larger role in conduction velocity than excitability or cell size [17,19,20]. Therefore, an enlarged atrium would lead to a larger distance traveled (D), and changes in connexin distribution and longitudinal conduction velocity, resulting in a longer flutter cycle length.  The degree that fibrosis uncouples gap junctions will vary between patients.  In patients with dilated atria and mild fibrosis atrial size is likely to be a very important determinant of flutter cycle length. When atrial fibrosis is severe, the effect of scarring on gap junctions (decreasing conduction velocity) may outweigh the impact of atrial dilatation (D increases < V decreases). Such differences account for the absence of linear correlation between right atrial size and flutter cycle length.

Rate-related historical definitions of typical isthmus dependent right atrial flutter have obvious limitations and indications for catheter ablation of the cavotricuspid isthmus should be expanded accordingly. Careful evaluation of the 12-lead electrocardiogram combined with the right atrial CSA will help identify patients likely to benefit from isthmus ablation, despite atrial rates below 250 bpm [21].

### Study Limitations

We excluded patients on antiarrhythmic medications known to affect conduction velocity and the atrial refractory period. In addition, all patients were supine, sedated and in the post-absorptive state, thus the interplay of volume loading and autonomic tone should be uniformly distributed in both groups under analysis. However, since echocardiography and electrophysiologic data collection were performed at different times, autonomic tone and/or volume loading could have been different at these two time points, accounting for some variability in individual patients' atrial rate data. Given the limitations of obtaining a true cross sectional area, our estimates may underestimate the true right atrial size. A better measure of the distance the wavefront of activation travels may require calculating an atrial perimeter. Finally, while the importance of conduction velocity in determining cycle length is well known, this measurement is difficult to obtain in vivo.

## Conclusions

The cycle length of typical atrial flutter is the result of a complex interaction between autonomic tone, atrial volume loading, effects of drugs on the excitable gap and intraatrial conduction time [2-5,22]. A well described correlation exists between slower right atrial flutter rates and Class I and III antiarrhythmic medication use. We have demonstrated a correlation between flutter cycle length and right atrial CSA. It is, therefore, likely that right atrial size has a significant impact on atrial flutter circuit wavelength. The non-linearity of the correlation between right atrial CSA and cycle length suggest that the multitude of structural and cellular factors that impact atrial impulse conduction velocity may have potential to overshadow the effect of right atrial CSA on flutter cycle length.

A well validated measurement of right atrial volume does not currently exist without expensive imaging (CT, MRI). Right atrial length and width measurements are readily available in most cardiology offices and hospitals. CSA calculation is simple, inexpensive and may be a useful tool in clinical arrhythmic evaluation.

## Figures and Tables

**Table 1:**
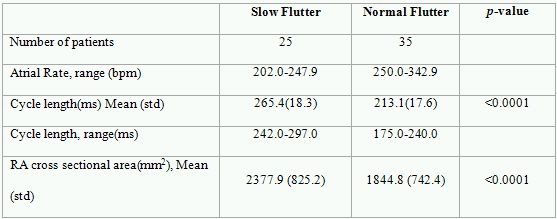


Avg - average, bpm-beats per minute, mm-millimeters, ms-milliseconds, RA-right atrium, std - standard deviation

## Abbreviations

Avg: Average
bpm: beats per minute
CSA: Cross-sectional area
LAFB: left anterior fascicular block
CT: Computerized tomography
mm: millimeters
ms: milliseconds
MRI: Magnetic Resonance Imaging
RA: Right Atrium
